# Upper Extremity Motor Learning among Individuals with Parkinson's Disease: A Meta-Analysis Evaluating Movement Time in Simple Tasks

**DOI:** 10.1155/2012/589152

**Published:** 2011-12-05

**Authors:** K. Felix, K. Gain, E. Paiva, K. Whitney, M. E. Jenkins, S. J. Spaulding

**Affiliations:** ^1^Faculty of Health Sciences, The University of Western Ontario, London, ON, Canada N6A 3K7; ^2^Clinical Neurological Sciences, Schulich School of Medicine and Dentistry, The University of Western Ontario, London, ON, Canada N6A 5C1; ^3^Faculty of Health Sciences, School of Occupational Therapy, The University of Western Ontario, London, ON, Canada N6G 1H1

## Abstract

Motor learning has been found to occur in the rehabilitation of individuals with Parkinson's disease (PD). Through repetitive structured practice of motor tasks, individuals show improved performance, confirming that motor learning has probably taken place. Although a number of studies have been completed evaluating motor learning in people with PD, the sample sizes were small and the improvements were variable. The purpose of this meta-analysis was to determine the ability of people with PD to learn motor tasks. Studies which measured movement time in upper extremity reaching tasks and met the inclusion criteria were included in the analysis. Results of the meta-analysis indicated that people with PD and neurologically healthy controls both demonstrated motor learning, characterized by a decrease in movement time during upper extremity movements. Movement time improvements were greater in the control group than in individuals with PD. These results support the findings that the practice of upper extremity reaching tasks is beneficial in reducing movement time in persons with PD and has important implications for rehabilitation.

## 1. Introduction

Motor learning is defined as a relatively permanent change in the ability to move associated with either practice or experience [1]. In neurologically healthy adults, brain activity changes occur in the basal ganglia during the process of motor learning [2]. From functional MRI studies, the key changes include a reduction of overall brain activation and a shift from cortical to more basal ganglia activity during the consolidation phase of learning [2, 3].

Parkinson's disease (PD) is a neurodegenerative disorder affecting basal ganglia functioning, characterized by four cardinal signs; bradykinesia (slowness of movement), rigidity (stiffness), resting tremor, and postural instability. Bradykinesia is an inherent component of PD and affects both movement initiation and execution [4, 5]. Motor deficits are not the only problem in PD. Due to the dysfunction of the basal ganglia in PD, motor learning may also be impaired.

Acquisition and retention of movement skills are important to researchers and clinicians who are involved in rehabilitation of individuals with PD [2, 6–8]. Nieuwboer et al. (2009) [6] reviewed 11 studies that evaluated acquisition and retention in a broad range of tasks. The studies suggest that overall, acquisition does occur in people with PD, but performance on the task during acquisition is typically impaired relative to controls. Nieuwboer et al.'s [6] review also suggests that long-term retention of new skills is impaired in individuals who have striatal problems, particularly in people with PD.

Although a number of studies have examined acquisition and retention of tasks in PD, the sample sizes have been small and heterogeneous, and the experimental tasks and outcomes used have varied widely. For example, kinematic variables, including distance (or displacement, which is distance with a specific direction), speed (or velocity, which is speed with a direction), and acceleration, have been used to measure motor learning both in the upper and the lower extremities in individuals with PD [9, 10]. Other movement parameters that have been measured include time, force, accuracy of movement to a target, coordination of more than one joint segment of the limb, sequencing of movement [9], interlimb function [11], and the ability to switch motor tasks [12]. Any of these measurements can provide researchers with valuable information about motor learning abilities in individuals with PD.

Regardless of the design features of each study, practice of the experimental task is integral to any of the research paradigms. While some researchers have suggested that people with PD do improve with practice, but not to the same level or as well as do control subjects [13–15], others have suggested that people with PD were able to benefit from short-term, but not long-term practice [16]. Sequence learning (learning of movements in a set sequence) has been shown to take more time and to be related to the stage of disease [13].

 Given the apparent heterogeneity of methodologies and participant samples, it is not surprising that there is disagreement on the extent and duration of skill acquisition in persons with PD. Such disagreement makes it difficult to draw firm conclusions and provide therapeutic recommendations to clinicians. To date, there have been systematic reviews, but no meta-analyses pooling or combining the existing data on acquisition and retention of skills in individuals with PD that may provide insight into the consistent effects of motor task practice.

 By focusing only on upper extremity and on movement time during practice of upper extremity reaching tasks, we were able to find a sufficient body of literature to analyze using a meta-analysis paradigm. The purpose of this study, therefore, was to determine how practicing a simple upper extremity motor task affects movement time for the task in people with PD.

## 2. Methods

### 2.1. Literature Search

The electronic databases used to find research that evaluated upper extremity motor learning in people with PD were CINAHL, EMBASE, PubMED, MEDLINE, PEDro, Proquest, PsycINFO, the Cochrane Database of Systematic Reviews, and Scopus. The comprehensive search used terms within the following categories: motor learning, Parkinson's disease, upper extremity, and time/speed/rate. The specific terms within categories are listed in Table 1.

The first four authors worked in pairs. Each pair was randomly assigned to search a set of databases and to select articles for screening. This initial search strategy resulted in 127 articles.

### 2.2. Criteria for Inclusion in Systematic Review

Once the set of 127 articles was retrieved, the first four authors evaluated them. The title, abstract, and full content of all articles were screened against the inclusion criteria, with each article appraised by two of the first four authors. Based on the criteria, articles for inclusion in the meta-analysis were chosen. Where there was disagreement between members of the pair of reviewers, the fifth and sixth authors (S. J. Spaulding and M. E. Jenkins) were consulted, and a consensus was reached. Inclusion criteria were as follows: articles that were published between the beginning of included databases up to September 2010, articles published in English, studies that examined upper extremity motor learning in individuals with PD, studies that included means and standard deviation or standard error, studies that evaluated motor learning with time as an outcome measure, and studies that had a control group.

Following the methodologies used by Siegert et al. [17], articles in the “grey literature,” such as conference proceedings or research published in Master's or PhD theses, were excluded to avoid the use of evidence that had not been peer reviewed at the level of a journal article. After the application of the initial inclusion criteria, the authors had determined that 30 articles met all the criteria.

The authors then examined the experimental design of these 30 articles to determine research that provided pre/postmeasurements of movement time prior to and following an intervention designed to elicit motor learning. The final group of articles included five publications published between 1998 and 2009. Within those articles, there were seven independent studies.

### 2.3. Data Extraction for Meta-Analysis

The first four authors working in pairs extracted the data from the seven independent studies. The following information was obtained for both experimental and control groups in all studies: sample size, pretraining mean, pretraining standard deviation or standard error, posttraining mean, and posttraining standard deviation. All time point values were documented immediately following the intervention and late (in terms of time after practice) as defined by each individual study. Data were extracted from text or figures, depending on how each article presented the data. If the resultant data were presented in a figure, each author, in the original pair of authors, extracted values, thus two measures were taken from the figure. The final value used was an average of the two authors' extracted numbers. Three studies reported both immediate and follow-up scores. When more than one follow-up period was measured, the authors chose to use the longest interval between training and followup. For the purposes of this meta-analysis, this period was termed late after training. Platz et al. [4] and Marinelli et al. [18] included two separate studies in their articles. The studies had different numbers of participants and different paradigms; thus, the results were entered into the analysis separately.

### 2.4. Meta-Analysis

A meta-analysis was conducted using the program Comprehensive Meta-Analysis (CMA) [19]. Hedge's *g*, a measure of the standardized mean difference, was determined for the pre/postscores in each of the control group and the group of individuals with PD. Hedge's *g *accounts for the overestimation of the population-standardized differences [20].

Because it could not be assumed that the people in the studies were highly homogeneous in their characteristics, a random effects model was used and provided a conservative estimate of the differences between the groups in the individual studies [20].

## 3. Results

A total of 58 individuals with PD and 56 participants without PD were included from the seven studies. Descriptive statistics of all the subjects are included in Table 2. Descriptive statistics of the findings extracted from the studies included in this meta-analysis are shown in Table 3.  Table 4 outlines the description of the motor learning paradigms in the studies used in the meta-analysis.

Hedge's *g* with a 95% confidence interval (CI) for each of the included studies is summarized in Table 5.

**Figure 1:**
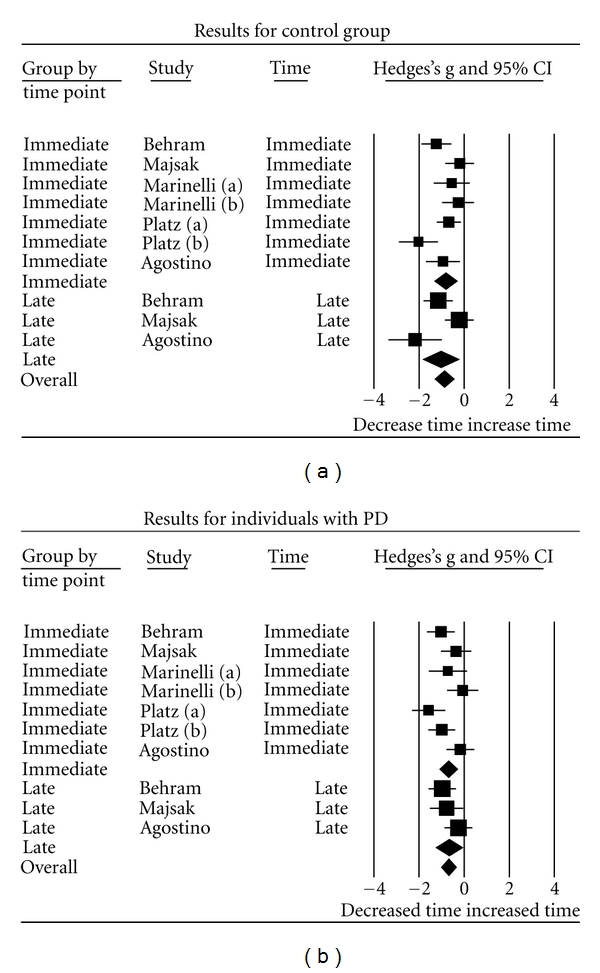
Forest plots of all the included studies for the control group (a) and the individuals with PD (b) including the time the results were acquired, Hedge's g, and 95% confidence interval (CI) for the control group. Each box and corresponding horizontal line represents the overall mean and confidence intervals in the movement time. The area of each box is proportional to the inverse of that study's variance. The horizontal line represents the 95% CI for each individual study. A diamond is used to depict overall mean effect size (center of the diamond) along with its CI (width of the diamond) [20].

As seen in the forest plot representing the results for the control group (Figure 1(a)), the point estimator of the overall effect shows that participants without PD demonstrated improvements in movement time. The point estimator of the overall effect for individuals with PD did show improvements, but the changes were smaller and showed greater variability than did the results of the control group (Figure 1(b)). The interval estimators of the overall effects (95% CI) for each group overlapped. When comparing movement times immediately (early) posttraining to late posttraining, slower times of movement and larger 95% CI were evident for the later posttraining time, for both groups.

## 4. Discussion

Although many studies have reported that motor learning occurs in individuals with PD, not all studies have reported improvements [4]. Among studies that examine the acquisition and retention of motor skills in PD, study sizes have been small, making conclusions less certain [6, 15]. In addition, tasks, duration of practice, and frequency of practice trials are different between studies [6]. This meta-analysis was able to overcome the heterogeneity issue by focusing only on studies of upper extremity movements and studies that analyzed improvements in movement time. Through the application of meta-analytic analysis, we were able to pool results with heterogeneous methods and demonstrate a consistent reduction in movement time as a result of practice of upper extremity reaching tasks.

The results of the meta-analysis suggest that motor learning in upper extremity function occurs in both neurologically healthy controls and individuals with PD through practice of upper extremity reaching tasks designed to reduce movement time. This effect is present immediately after the training period but also is sustained after a period of time although the late effects are somewhat diminished. The control participants have a mild to moderate increased effect based on their mean effect sizes compared to people with PD. However, the substantial overlap of confidence intervals would suggest that both groups benefit from the practice in which they participate.

Overall, these results are consistent with previous work in small studies that demonstrate skill acquisition and retention in people with PD in a variety of motor tasks. Such studies have demonstrated acquisition and retention of motor skills in varied upper extremity tasks not included in this meta-analysis such as serial reaction time tasks [25–27] and other sequential aiming movements [7, 9, 13]. Furthermore, motor learning studies in people with PD have demonstrated improvement in balance and lower extremity function through practice [10, 28–30].

In addition, motor learning effect, demonstrated by improvement in movement time, was smaller among individuals with PD. This is not particularly surprising, given the role of the basal ganglia in both acquisition of motor task skill and in consolidation of automatic movements [2, 3, 31]. As evidence of the potential alterations of brain activity in persons with PD during task learning, functional MRI studies in individuals with PD have demonstrated that greater areas of the brain are activated during initial learning of a task and particularly during the repetition of a learned movement in PD compared to healthy controls [31].

### 4.1. Rehabilitation Implications

A number of differences were identified in the experimental methodologies of the studies from which data were extracted to conduct this meta-analysis. There was variability among the duration and frequency of practice as well as the types of tasks. These differences preclude the authors from determining that there is one type of practice that was more effective to improve upper extremity performance. However, one can conclude that practice in general is beneficial and the manipulation of practice parameters is worthy of further study. Interestingly, even in the studies in which the individuals were off dopamine replacement medication [4, 18], there was a decrease in movement time, suggesting that there could potentially be a rehabilitation program that would benefit people with PD, even if medication effectiveness was suboptimal for some reason. Yet, current studies suggest that dopamine replacement medication may have a deleterious effect on motor learning [32].

### 4.2. Limitations of the Study

A limitation of the present meta-analysis is the small number of studies that the authors were able to include, but to the best of our knowledge, all of the available studies of simple reaching tasks reporting movement time as an outcome were incorporated. There are more studies evaluating practice, but they were heterogeneous in their tasks or in their outcome measures; therefore, they did not meet our inclusion criteria, and the data could not be included in this meta-analysis. Additionally, the sample sizes of the included studies were small, affecting the generalizability of this meta-analysis [33].

### 4.3. Recommendations for Future Research

Current literature in this area typically examines one single task or movement. Future research might best examine the generalizability of the effects of practice to other tasks and areas of rehabilitation. Conclusions from a broader range of tasks could lead to the use of programs that are directly related to movements needed for daily functioning. Finally, future motor skill acquisition research should further examine the effects of varied practice parameters in more diverse samples of persons with PD.

## 5. Conclusions

Results from this pooling of data from various studies provide evidence that upper extremity movement time can be improved through the use of practice of reaching tasks in persons with PD, albeit potentially to a lesser extent than is shown in individuals with no neurological problems. The collective interpretation of this meta-analysis indicates that practice of relevant motor tasks targeted at maximizing acquisition and retention improved movement speed.

## Figures and Tables

**Table 1:** Search terms used for the meta-analysis.

	Parkinson's disease	Upper extremity	Time/speed/rate
Practice	Parkinson	Arms	Reaction time
Training	PD	Upper limb	Serial reaction time
Sequential learning	Parkinson's	Hand	Reach time
Procedural learning	Parkinson disease	Wrist	Hand to mouth time
Motor skill learning		Reaching	Movement time
Skill learning			Response time
Task performance			Reaction speed
Task demand			Serial reaction speed
Response programming			Reach speed
Motor function			Hand to mouth speed
Motor function loss			Movement speed
Motor activity			Response speed
			Reaction rate
			Serial reaction rate
			Reach rate
			Hand to mouth rate
			Movement rate
			Response rate

**Table 2:** Descriptive statistics of participants with PD in the included studies.

Study	Age mean (SD)	MMSE mean (SD)	Duration of PD in years mean (SD)	Hoen and Yahr stage mean (SD)	UPDRS mean (SD)	Medication status (related to anti-Parkinsonian medication)
Agostino et al. (2004) [21]	64.4 (6.3)	>26	7.6 (3.1)	N/A^1^	15.3 (4) (motor score)	On
Behrman et al. (2000) [22]	74 (7)	28 (1.6)	7 (4)	2.6 (0.5)	N/A^1^	On
Majsak et al. (2008) [23]	70.4 (3.7)	N/A	7.3 (7.9)	3 (0)	33 (7.5) (motor score)	On
Marinelli et al. (2009)^a^ [18]	60 (7.4)	≥27	8.4 (4.5)	2 to 2.5	N/A^1^	On
Marinelli et al. (2009)^b^ [18]	57.9 (7.3)	≥27	2.1 (3.1)	1 to 2	N/A^1^	Off
Platz et al. (1998)^a^ [4]	65.9 (8.3)	27.7 (1.6)	7.6 (2.4)	2.5 (0.5)	8.0 (4) Bradykinesia score^2^	Off
Platz et al. (1998)^b^ [4]	62.0 (14.6)	28.8 (1)	4.3 (1.8)	2.0 (.75)	4.0 (3.5) Bradykinesia score	Off

^1^N/A indicates that the results were not available. SD: standard deviation.

^2^[24].

Note: ^a^ and ^b^ are data from two different paradigms within one publication.

^c^ and ^d^ are data from two different experiments within one publication.

**Table 3:** Descriptive statistics of studies of upper lime reach task.

Study	Control group			Parkinson's disease		
	Pre	Immediate post	Late post	Pre	Immediate post	Late post
	Mean time (SD) units: seconds			Mean time (SD) units: seconds		
Agostino et al. (2004) [21] *N* = 9 (PD) *N* = 7 (controls)	0.305 (0.026)	0.271 (0.035)	0.238 (0.246)	0.325 (0.286)	0.275 (0.750)	0.250 (0.394)
Behrman et al. (2000) [22] *N* = 15 (PD) *N* = 15 (controls)	0.183 (0.068)	0.106 (0.038)	0.111 (0.041)	0.200 (0.074)	0.130 (0.032)	0.134 (0.035)
Majsak et al. (2008) [23] *N* = 8 (PD) *N* = 8 (controls)	0.388 (0.062)	0.375 (0.058)	0.375 (0.035)	0.547 (0.110)	0.505 (0.095)	0.463 (0.047)
Marinelli et al. (2009)^a^ [18] *N* = 5 (PD) *N* = 5 (controls)	0.440 (0.014)	0.430 (0.015)		0.440 (0.011)	0.430 (0.011)	
Marinelli et al. (2009)^b^ [18] *N* = 11(PD) *N* = 11 (controls)	0.425 (0.027)	0.415 (0.035)		0.400 (0.023)	0.415 (0.189)	
Platz et al. (1998)^c^ [4] *N* = 7 (PD) *N* = 7 (controls)	0.750 (0.138)	0.550 (0.072)		0.950 (0.051)	0.850 (0.080)	
Platz et al. (1998)^d^ [4] *N* = 8 (PD) *N* = 8 (controls)	0.750 (0.138)	0.620 (0.072)		0.950 (0.051)	0.865 (0.080)	

*N*: number of subjects in each group, SD: standard deviation.

Note: ^a^ and ^b^ data were extracted from two different paradigms within one publication. The first paradigm did not include cueing and the second did.

^c^ and ^d^ data were extracted from two different experiments within one publication.

**Table 4:** Description of the motor learning paradigms in the studies used in the meta-analysis.

Study	Type of task	Duration of practice	Frequency of practice trials
Agostino et al. (2004) [21]	Visually guided motor sequence in free space.	100 motor sequences trials.	1 session/day (Monday to Friday). 2 weeks of 5 sessions/week.
Behrman et al. (2000) [22]	Two simple sequential arm-reaching tasks between targets 12.7 cm apart.	120 reaction time trials.	1 session on each of 2 days.
Majsak et al. (2008) [23]	Reaching a ball in front of person.	5 blocks of 4 trials with blocks of stationary, moving, or drop ball conditions.	90 minutes, approximately. 1 session.
Marinelli et al. (2009)^a^ [18]	Reach on digitized tablet to a rotating target from center.	48-second blocks of two tasks: with and without rotation.	1 session.
Marinelli et al. (2009)^b^ [18]	Reach on digitized tablet. Counterclockwise predicted. Clockwise not predicted.	90-second blocks of each of two tasks: predictable and unpredictable.	1 session.
Platz et al. (1998)^c^ [4]	Pointing from starting position to target 20 cm away.	15 trials baseline, 100 trials practice, and 15 trials with each limb.	1 session.
Platz et al. (1998)^d^ [4]	Pointing from starting position to target 20 cm away. Timing cues provided.	15 trials baseline, 100 trials practice, and 15 trials with each limb.	1 session.

Note: ^a^ and ^b^ are data from two different paradigms within one publication.

^c^ and ^d^ are data from two different experiments within one publication.

**Table:** (a)

Authors		Control group		Individuals with PD	
	Time of testing*	Effect size (Hedge's *g*)	95% CI	Effect size (Hedge's *g*)	95% CI
Agostino et al. (2004) [21]	Immediate	−0.937	−1.668 to −0.582	−0.177	−0.773 to 0.419
Behrman et al. (2000) [22]	Immediate	−1.233	−1.884 to −0.582	−1.031	−1.663 to −0.426
Majsak et al. (2008) [23]	Immediate	−0.192	−0.815 to 0.431	−0.361	−1.002 to 0.280
Marinelli et al. (2009)^a^ [18] experiment 1	Immediate	−0.551	−1.331 to 0.229	−0.727	−1.561 to 0.106
Marinelli et al. (2009)^b^ [18] experiment 2	Immediate	−0.265	−.955 to 0.425	−0.071	−0.746 to 0.604
Platz et al. (1998)^c^ [4] study 1	Immediate	−0.667	−1.197 to −0.156	−1.581	−2.400 to −0.863
Platz et al. (1998)^d^ [4] study 2	Immediate	−2.030	−2.873 to −1.186	−0.992	−1.571 to −0.414

Group immediate effect		−0.814	−1.288 to −0.340	−0.698	−1.070 to −0.325

**Table:** (b)

Authors		Control group		Individuals with PD	
	Time of testing*	Effect size (Hedge's *g*)	95% CI	Effect size (Hedge's *g*)	95% CI

Agostino et al. (2004) [21]	Late	−2.174	−3.339 to −1.009	−0.256	−0.857 to 0.346
Behrman et al. (2000) [22]	Late	−1.148	−1.778 to −0.517	−0.973	−1.565 to −0.381
Majsak et al. (2008) [23]	Late	−0.215	−0.839 to 0.410	−0.781	−1.506 to −0.056

Group late effect		−1.028	−1.784 to 0.272	−0.665	−1.226 to −0.105

Overall effect^e^		−0.875	−1.276 to −0.473	−0.688	−0.998 to −0.377

Note*:* ^a^ and ^b^ data were extracted from two different experiments within one publication. ^c^ and ^d^ data were extracted from two different training programs within one publication. Effect size was corrected using Hedge's *g*.

^e^The overall effect is the combination of the group immediate effect and the group late effect.

*Time of testing is indicated as either immediately following training (immediate) or following an interim period specified by each individual study (late).
